# Th17 Cells and Activated Dendritic Cells Are Increased in Vitiligo Lesions

**DOI:** 10.1371/journal.pone.0018907

**Published:** 2011-04-25

**Authors:** Claire Q. F. Wang, Andres E. Cruz-Inigo, Judilyn Fuentes-Duculan, Dariush Moussai, Nicholas Gulati, Mary Sullivan-Whalen, Patricia Gilleaudeau, Jules A. Cohen, James G. Krueger

**Affiliations:** 1 Laboratory for Investigative Dermatology, The Rockefeller University, New York, New York, United States of America; 2 Weill Cornell Medical College, New York, New York, United States of America; Universität Würzburg, Germany

## Abstract

**Background:**

Vitiligo is a common skin disorder, characterized by progressive skin de-pigmentation due to the loss of cutaneous melanocytes. The exact cause of melanocyte loss remains unclear, but a large number of observations have pointed to the important role of cellular immunity in vitiligo pathogenesis.

**Methodology/Principal Findings:**

In this study, we characterized T cell and inflammation-related dermal dendritic cell (DC) subsets in pigmented non-lesional, leading edge and depigmented lesional vitiligo skin. By immunohistochemistry staining, we observed enhanced populations of CD11c+ myeloid dermal DCs and CD207+ Langerhans cells in leading edge vitiligo biopsies. DC-LAMP+ and CD1c+ sub-populations of dermal DCs expanded significantly in leading edge and lesional vitiligo skin. We also detected elevated tissue mRNA levels of IL-17A in leading edge skin biopsies of vitiligo patients, as well as IL-17A positive T cells by immunohistochemistry and immunofluorescence. Langerhans cells with activated inflammasomes were also noted in lesional vitiligo skin, along with increased IL-1ß mRNA, which suggest the potential of Langerhans cells to drive Th17 activation in vitiligo.

**Conclusions/Significance:**

These studies provided direct tissue evidence that implicates active Th17 cells in vitiligo skin lesions. We characterized new cellular immune elements, in the active margins of vitiligo lesions (e.g. populations of epidermal and dermal dendritic cells subsets), which could potentially drive the inflammatory responses.

## Introduction

Vitiligo is a common skin disorder, affecting over 0.5% of the world population [1]. It is characterized by progressive skin de-pigmentation due to the loss of cutaneous melanocytes and abnormal melanocyte function. There are two types of vitiligo: segmental and non-segmental. Non-segmental vitiligo occurs at sites sensitive to pressure or friction, and it accounts for up to 90% of cases overall [2]. The exact cause of melanocyte loss in non-segmental vitiligo is still debatable, but many observations have pointed to the important role of cellular immunity in its disease pathogenesis. [3], [4], [5], [6].

Earlier studies have shown that depigmenting vitiligo skin is accompanied by lymphocytic infiltrates containing both CD4+ and CD8+ T cells at the dermal-epidermal junction. The skin-infiltrating cytotoxic T cells were found to be juxtaposed with melanocytes and were enriched for melanocyte antigen recognition [7], [8]. T cells isolated from peri-lesional skin of vitiligo patients also showed cytotoxicity against autologous melanocytes *in vitro* [9]. The onset of vitiligo seen following immunotherapy of melanoma using infusion of Melan-A specific CD8+ T-cell clones or dendritic cell vaccines provide additional support for the autoimmune hypothesis of vitiligo pathogenesis [10], [11].

Like in many other inflammatory diseases, the pathogenesis of vitiligo includes an active population of T-helper 1 cells [12], [13], and the treatment of vitiligo using IFN-γ inhibition has given positive responses [14]. Although Th17 polarization is another important arm in the progression of inflammatory diseases including psoriasis [15], [16], [17] [18], [19], there have been very limited data on whether Th17 cells participate in the pathogenesis of vitiligo [20].

In this study, we aimed to characterize the Th1 and Th17 components in vitiligo, as well as the epidermal Langerhans cell and myeloid dermal dendritic cell populations, which are capable of driving the proliferation of T cells by presenting autoantigens and producing inflammatory cytokines [19], [21]. It has been demonstrated that Langerhans cells and myeloid dermal dendritic cells can stimulate T cells to directly expand Th1, Th2 and Th17 responses [22], [23], [24], [25]. Hence, one can take the view that autoinflammatory or autoimmune responses in the skin can be driven by factors that, in focal skin regions, will activate DCs, which might then activate specific T cell populations in the skin.

## Results

### Loss of melanocytes in lesional and leading edge vitiligo skin

**Figure 1:**
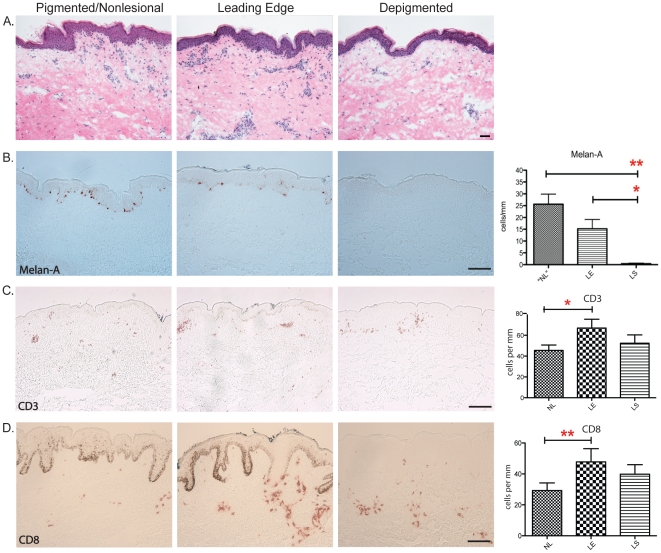
T cell infiltration and loss of melanocytes in lesional and leading edge vitiligo skin. (A) Hematoxylin and eosin staining of non-lesional, leading edge and depigmented vitiligo skin does not reveal any abnormality, but significant immune cell infiltrations were found in the dermal regions of leading edge vitiligo biopsies. Bar = 100 mm. (B) Immunohistochemical staining of Melan-A on non-lesional, leading edge and depigmented vitiligo skin. The staining pattern of Melan-A is consistent with the degree of depigmentation in our vitiligo biopsies. Bar = 100 mm. Bar charts to the right of (B) is cell count of Melan-A positive cells of non-lesional, depigmented lesional and leading edge skin in biopsies of vitiligo patients. Non-lesional vitiligo skin has significantly higher number of melanocytes than lesional (p<0.0027) and leading edge skin (p<0.0082). Leading edge has significantly higher numbers of Melan-A positive cells than lesional vitiligo skin (C) Immunohistochemical staining of CD3+ T cells at non-lesional, leading edge and depigmented lesional vitiligo skin. (D) Immunohistochemical staining of CD8+ T cells at non-lesional, leading edge and depigmented lesional vitiligo skin. Bar = 100 mm, applies to both C&D. T cell infiltrates are predominantly found at the dermal-epidermal junctions in the leading edge vitiligo skin. Bar charts on the right to C&D provide the numbers from quantitative cell counting of CD3+ and CD8+ T cells, respectively.

Vitiligo is characterized by the loss of melanocytes and the resulting absence of melanin from the epidermis [2]. Here, we labeled Melan-A, a melanocyte marker, to quantify melanocytes in normal appearing non-lesional skin, depigmented lesional skin and the leading edge skin from vitiligo patients. Melan-A is a melanosomal protein that can be recognized by autologous cytotoxic T lymphocytes [26], [27], [28]. In Fig. 1B, immunohistochemical staining of Melan-A showed abundant expression in pigmented non-lesional skin. Melanized keratinocytes and melanocytes were found at the dermal-epidermal junction. By comparison, staining of the leading edge of depigmented vitiligo skin showed fewer Melan-A positive cells (*p*<0.0956). In depigmented lesional skin samples, melanocytes were absent and no positivity was found (*p*<0.0082). The staining pattern of Melan-A shown in Fig. 1B coincides with the degree of depigmentation in our vitiligo biopsies.

### T cell infiltration at lesional and leading edge vitiligo skin

Previous reports of T cell infiltration accompanying vitiligo progression have suggested T cell mediated cytotoxicity as a mechanism for melanocyte killing [29], [30]. As expected, significant CD3+ and CD8+ T cell infiltration was observed in vitiligo skin, and clusters of T cells were identified near the disappearing melanocytes at dermal-epidermal junctions (Fig. 1C&D). These T cells predominately infiltrated to the leading edge of depigmented skin, where progressive loss of skin pigmentation and destruction of melanocytes were taking place. CD3+ T cells were localized primarily in the papillary and upper reticular dermis of the leading edge of vitiligo lesions and were often organized as aggregates (Fig. 1C). Another major distinction between leading edge vitiligo skin and non-lesional skin is that T cells were frequently observed to be in direct contact with the basal epidermis or infiltrating the epidermis in the former. Double fluorescence stainings of CD3/CD4 and CD3/CD8 are included as Figure S1&S2. In the skin, a majority of CD3+ cells are also CD4+ (>60%). CD3−/CD4+ population will include dermal dendritic cells; CD3+/CD4− cells are also detected, indicating a mixed CD4+ and CD8+ T cell infiltrate in vitiligo.

### Analysis of DC subsets in vitiligo skin

**Figure 2:**
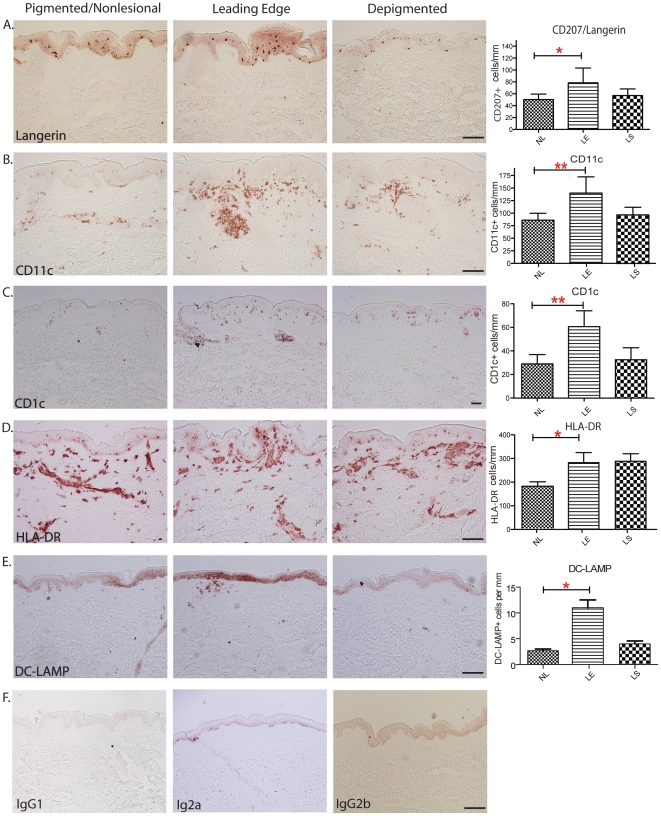
Characterization of Langerhans cells, dermal dendritic cells subpopulations in matched non-lesional, leading edge and depigmented lesional skin. Quantification of each cell population per mm of skin appears on the right side of the micrograph image panels. Representative immunohistochemical staining on non-lesional, leading edge and depigmented lesional skin of (A) CD207/Langerin: Langerhans cell marker; (B) CD11c: myeloid dermal dendritic cell marker; (C) CD1c/BDCA-1: resident dermal dendritic cell marker; (D) HLA-DR: Activated Langerhans cell and mature dermal DC marker. (E) DC-LAMP: mature DC marker. (F) Mouse IgG1, IgG2a, and IgG2b were applied at the same concentrations as the matching primaries antibodies on leading edge vitiligo skin. Bar = 100 mm, applies to 2A, B, C, D&E.

Langerhans cells were quantified by CD207/Langerin staining (Fig. 2A). Quantitative image analysis by cell counting showed that compared to non-lesional skin, the leading edge contained higher numbers of epidermal Langerhans cells (*p*<0.0339). In leading edge and lesional biopsies, Langerhans cells tended to reside in the lower half of the epidermis. However, in the uninvolved skin of these patients, Langerhans cells were more uniformly distributed in the stratum malpighii of the epidermis, which is more similar to their distribution pattern in normal skin of healthy individuals.

We used integrin CD11c as a general marker for quantifying dermal dendritic cell populations across non-lesional, lesional, and leading edge vitiligo skin biopsies. In normal skin, CD11c+ DCs are found in the papillary and upper reticular dermis, but direct contact with basal keratinocytes is not apparent [19]. In contrast, in vitiligo biopsies, CD11c+ myeloid DCs preferentially localized to the dermal-epidermal junction, often forming aggregates (Fig. 2B). In leading edge and lesional biopsies, CD11c+ DCs were frequently juxtaposed with basal keratinocytes/melanocytes, and some invasion of CD11c+ DCs into the basal epidermis was apparent. Leading edge biopsies contained significantly larger numbers of CD11c+ DCs than pigmented non-lesional (*p*<0.0183) and also lesional samples. Additionally, larger numbers of CD11c+ DCs were found in lesional skin than pigmented non-lesional vitiligo skin (*p*<0.0184).

One subset of CD11c+ dermal DCs is the CD1c/BDCA-1+ resident dermal DCs [23]. In normal skin, these CD11c+ CD1c+ cells are relatively immature with modest T-cell stimulatory ability. Here, the CD11c+ CD1c+ dendritic cells were found throughout the upper dermis of non-lesional, lesional and leading edge vitiligo skin (Fig. 2C). Clinically pigmented skin contained the lowest numbers of CD1c+ DCs, while leading edge biopsies showed the highest numbers of CD1c+ DCs (*p*<0.0588).

CD11c also marks another subset of CD11c+/CD1c− DCs, which we have termed “inflammatory dermal DCs”, or, in the case of psoriasis, “TIP-DCs” (TNF and iNOS-producing DCs) [21], [31]. In this study, we saw a remarkable rise in the number of CD11c+ DCs in the leading edge of vitiligo skin, which contained enhanced populations of both CD1c+ and CD1c− DCs (Fig. 2B). This result is different from the observations we made in other disease models, including psoriasis and squamous cell carcinoma, where the CD11c+ CD1c+ DC population stays relatively unchanged in number, but the CD11c+ CD1c− DC population shows a dramatic expansion [32].

### More mature/activated Langerhans cells and dermal DCs are found in leading edge vitiligo skin

We also stained for HLA-DR+ and DC-LAMP+ cells across the three groups of vitiligo skin biopsies (i.e. lesional, non-lesional, leading edge) (Fig. 2D&E). Our staining showed regular distributions of Langerhans cells in the suprabasal layers of the epidermis in almost all vitiligo biopsies, and the highest number of DC-LAMP expressing cells was found in leading edge skin. The expression of HLA-DR was higher on dendritic cells in the epidermis, suggesting that Langerhans cells in the depigmenting lesions are more activated compared to non-lesional skin (Fig. 2D). Increased numbers of HLA-DR+ cells were recorded in leading edge (p<0.0358) and lesional biopsies. Aggregates of DC-LAMP+ cells were seen at dermal-epidermal junctions in leading edge skin, and their numbers were noticeably higher than that of non-lesional (p<0.046) or depigmented lesional skin (Fig. 2E). Scattered CD83+ cells with dendritic morphology were found in the upper dermis and epidermis of vitiligo biopsies, including non-lesional, leading edge and depigmented. Some CD83+ cells are located in the epidermis, consistent with LC maturation It is important to note that normal skin does not contain this CD83+ cell population. (Figure S3). For negative controls, mouse IgG1, IgG2a and IgG2b were applied to leading edge vitiligo skin sections at the same concentration as primary antibodies of the same isotypes, and immunohistochemistry micrographs are shown in Fig. 2F. Prior to performing immunohistochemistry staining on vitiligo biopsies, all primary antibodies used in Fig. 2 were tested on psoriasis lesional and non-lesional skin for their reactivity and specificity (Fig. S4). The staining patterns of the antibodies listed in Table S1 on Langerhans cells and dermal DCs of psoriatic skin were consistent with data published in previous reports [19], [33].

### NALP-1 positive Langerhans cells are found in lesional vitiligo skin

**Figure 3:**
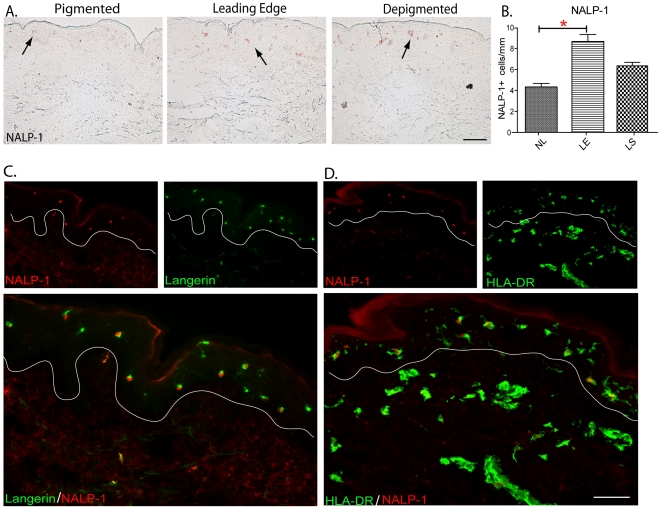
NALP-1 positive Langerhans cells are found in lesional vitiligo skin. Immunohistochemical staining of NALP-1 on vitiligo biopsies and double staining of NALP-1 with CD207 and HLA-DR on lesional skin. (A) Immunohistochemical staining shows higher numbers of NALP-1 positive inflammasomes in leading edge vitiligo skin biopsies when compared to normal skin from healthy patients. (B) Quantification of NALP-1 positive cells per mm of skin (p<0.05) (C) NALP-1 colocalized with Langerin/CD207, a Langerhans cell marker, in lesional vitiligo skin. (D) NALP-1 colocalized with HLA-DR, a marker for activated dendritic cells, in lesional vitiligo skin. In 3C&D, single-stained controls are above the merged image, white line denotes dermal-epidermal junction. Fluorescent signals of NALP-1 appear as red, whereas Langerin and HLA-DR signals appear as green. Areas of colocalization appear as orange. Bar = 100 mm, applies to both A, C&D.

NALP-1 is part of the cytoplasmic complexes called inflammasomes that regulate the activation of caspases, which in turn convert the proinflammatory cytokines (e.g. IL-1ß) into their active forms [34]. Ying Jin and colleagues showed that variants of NALP-1 may confer susceptibility to autoimmune and autoinflammatory diseases that are associated with vitiligo [35], [36]. Here, immunohistochemical staining of NALP-1 shows that the leading edge vitiligo biopsies contained higher numbers of NALP-1 positive epidermal cells compared to depigmented lesional and pigmented nonlesional skin (Fig. 3A&B, p<0.05). We also performed double immunofluorescence staining of NALP-1 and CD207/Langerin on vitiligo biopsies (Figure S5). NALP-1 co-localized with CD207/Langerin, the Langerhans cell marker, in the epidermal region of leading edge vitiligo skin. NALP-1 bearing Langerhans cells appear as orange dots in merged images (Fig. 3C), and more noticeable colocalization of NALP-1 and CD207/Langerin were found in leading edge vitiligo skin. Double immunofluorescence staining of NALP-1 and HLA-DR also showed higher numbers of activated HLA-DR^high^ Langerhans cells with positive NALP-1 expression in the epidermis of leading edge vitiligo skin (Fig. 3D).

### Cytokines defining Th1 and Th17 but not Th2 activation were increased in leading edge and lesional vitiligo skin

**Figure 4:**
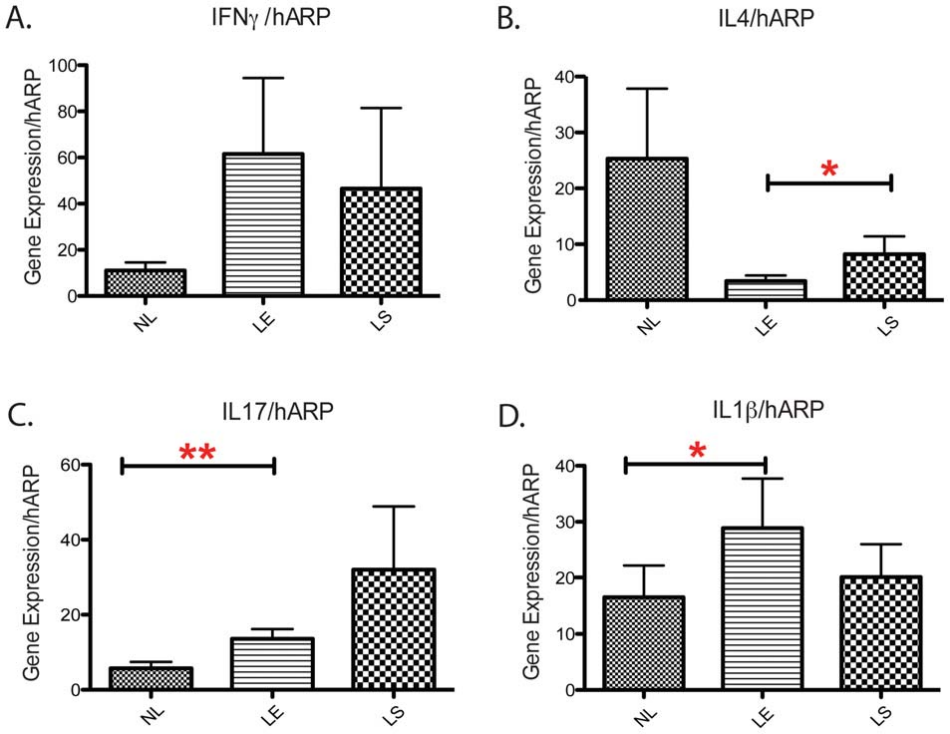
qPCR analysis of Th1, Th2 and Th17 cytokines in non-lesional, leading edge and lesional skin. Gene expression levels of key cytokines were adjusted against hARP across matching non-lesional, leading edge and lesional vitiligo skin pairs. (A) IFN-g and (B) IL-4 are hallmark cytokines for Th1 and Th2 polarization, respectively. (C) IL-17A and (D) IL-1ß serve as hallmark cytokines for Th17 polarization; Two way paired t-tests are performed for each data of gene expression data between non-lesional *v.s.* lesional, non-lesional *v.s.* leading-edge vitiligo skin. *p<0.05; **, *p*<0.01.

To compare the responses from different T helper cell subpopulations in our vitiligo patients, we assessed the levels of key cytokines: IFN-γ, IL-4 and IL-17A, which are hallmarks of Th1, Th2 and Th17 polarization, respectively. We also examined the levels of IL-1ß, an important cytokine for Th17 cell development [37]. High expression levels of IFN-γ were detected in leading edge vitiligo skin, indicating Th1 cell activity (p<0.0705) (Fig. 4A). In contrast, IL-4 levels were lowest in leading edge vitiligo skin, when compared to non-lesional and lesional skin (Fig. 4B).

Th17 polarization of T helper cells in vitiligo was assessed by qPCR analysis of mRNA levels of IL-17A. We found that IL-17A expression in the leading edge was consistently higher than non-lesional skin across different patient samples (p<0.0069) (Fig. 4C). In addition, IL-1ß mRNA levels were highest in leading edge skin biopsies, and the lowest expression level was found in non-lesional samples (p<0.0313) (Fig. 4D).

### Increased numbers of IL-17A+ cells and IL-17RA+ cells were found at the upper dermis of leading edge vitiligo skin

**Figure 5:**
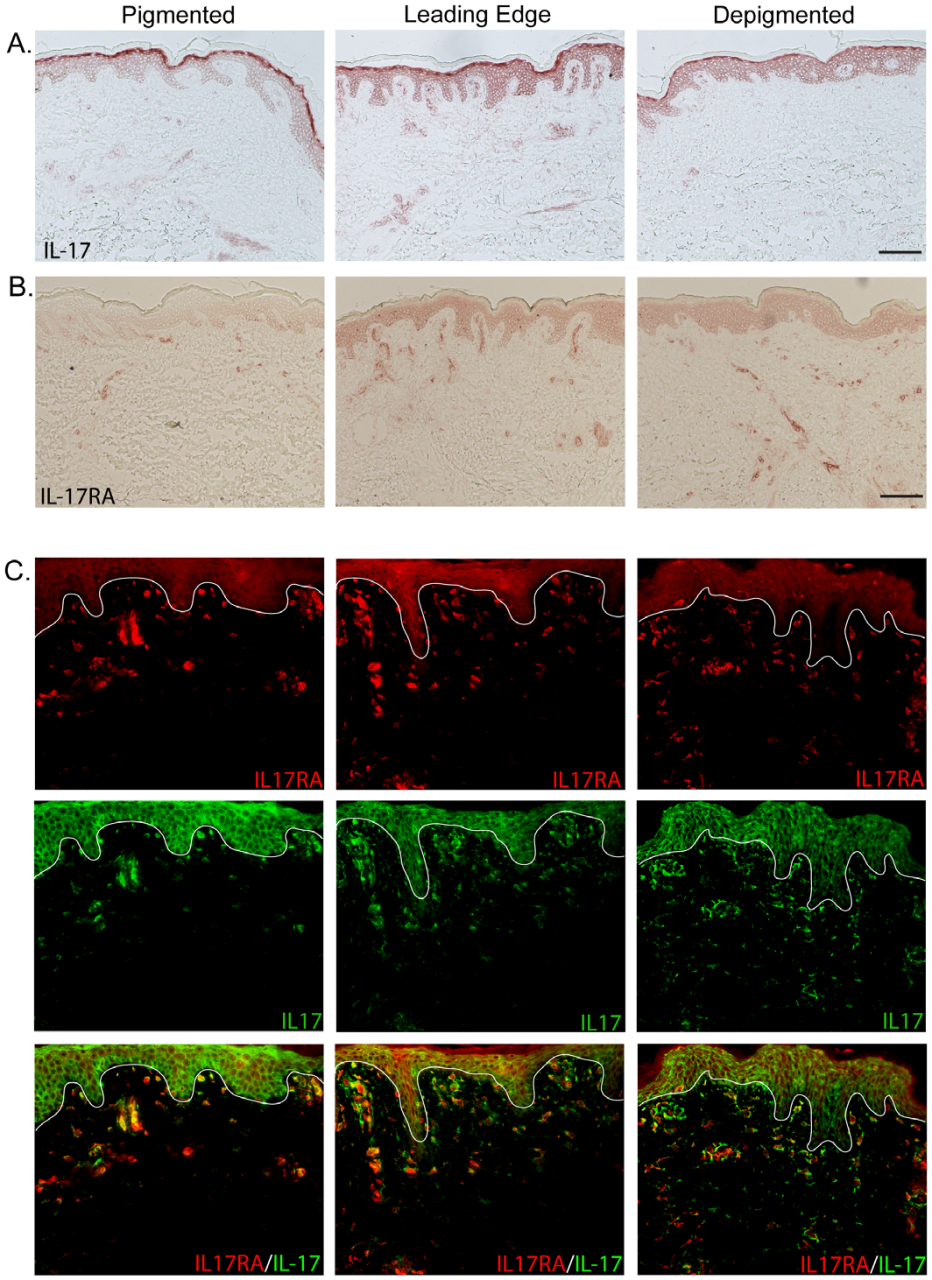
IL-17A and IL-17RA are found on vitiligo skin biopsies. Immunohistochemistry and immunofluorescence staining of IL-17A and IL-17 receptor A on vitiligo skin biopsies. (A)&(B) In immunohistochemistry, IL-17A and IL-17RA showed strong staining on the upper dermis of leading edge vitiligo skin when compared to non-lesional/pigmented vitiligo skin. (C) Double immunofluorescence staining of IL-17A and IL-17RA, areas of orange shows receptor bound IL-17A molecules. 50–60% of IL-17RA positive cells are also IL-17A positive in leading edge vitiligo biopsies. In comparison, only 20–30% of IL-17RA positive cells are IL-17A positive in non-lesional and lesional skin. Bar = 100 mm, applies to 5A, B&C.

To further verify our finding, we performed immunohistochemical staining of IL-17A and IL-17RA across the three groups of vitiligo biopsies. Leading edge vitiligo biopsies showed more IL-17A+ cells and IL-17RA+ cells in the upper dermis compared to non-lesional vitiligo skin (Fig. 5A&B). Double immunofluorescence staining of IL-17A and IL-17RA was performed to identify receptor bound IL-17A. We observed that 50–60% of IL-17RA positive cells are also IL-17A positive in leading edge vitiligo biopsies. In comparison, only 20–30% of IL-17RA positive cells are IL-17A positive in non-lesional and lesional skin (Fig. 5C). In order to verify the specificity of our IL-17A antibody, a blocking experiment using recombinant IL-17A was performed on psoriasis lesional and non-lesional skin biopsies. After pre-incubation with recombinant human IL-17A, no staining was observed except for precipitates of antigen/antibody complexes (Fig. S6). Psoriasis lesional skin was used as a positive control, since the involvement of the IL-17/IL-23 axis in psoriasis has been well characterized by several studies from this lab [19], [22], [33], [38].

### IL-17A producing T cells were found in vitiligo skin

**Figure 6:**
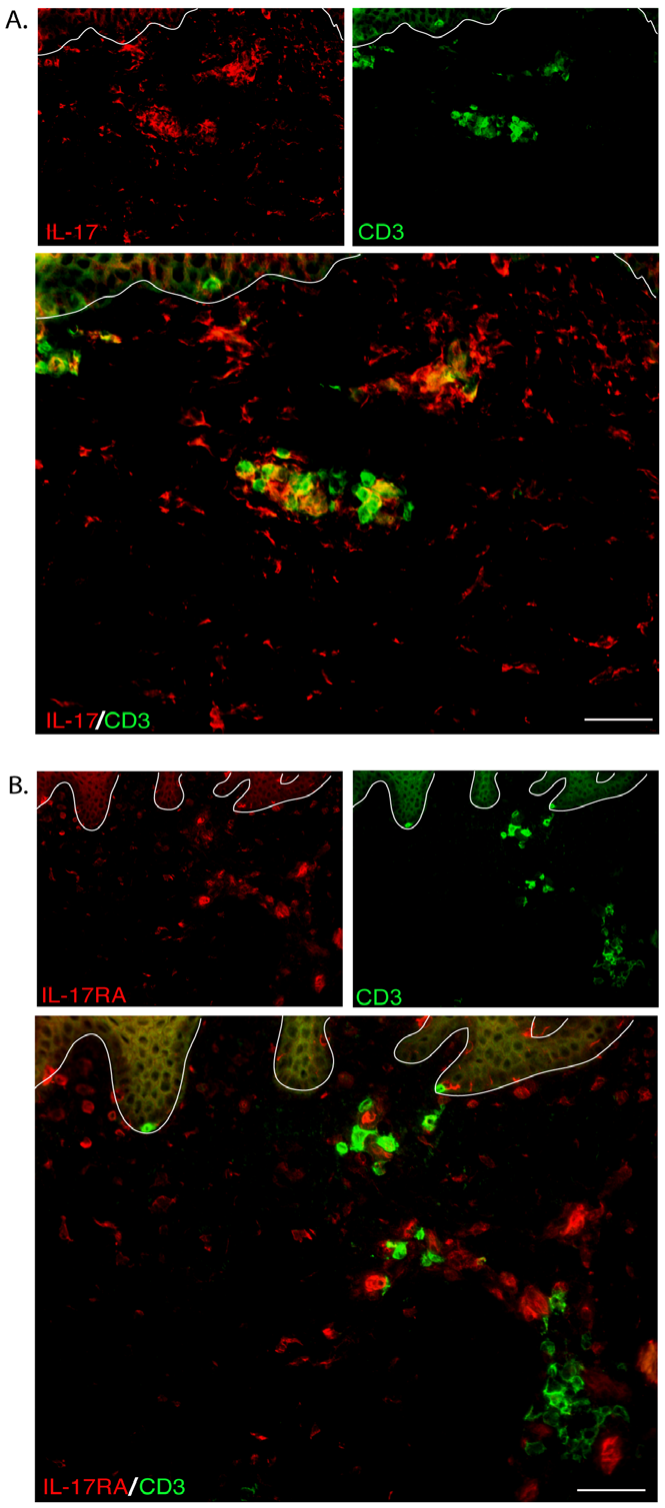
Detection of IL-17A producing T cells in vitiligo skin biopsies. Double immunofluorescent staining of IL-17A/IL-17RA with CD3 on leading edge vitiligo skin biopsies. (A) Co-staining of CD3 and IL-17A reveals IL-17A+ T cells in vitiligo skin biopsies, but overlapping signals were only found on a small number of CD3+ cells. IL-17A signals on CD3 negative cells may reflect IL-17A molecules that are receptor bound. (B) Co-staining of CD3 and IL-17RA does not show any overlapping signals across the three groups of vitiligo biopsies, which indicates that receptor bound IL-17A are not likely to colocalize with CD3+ T cells. Bar = 100 mm, applies to 6A&B.

Double immunofluorescence staining of IL-17A and CD3 in leading edge vitiligo skin biopsies showed clear colocalization, especially for CD3+ T cells that appeared as aggregates in the upper dermis (Fig. 6A). It is worthy to note that only less than 10% of CD3+ cells were also IL-17A positive. IL-17A staining was also seen on some CD3 negative cells, which may indicate NK cells and other types of cells that produce IL-17A. It is important to note that this staining pattern may actually reflect binding of IL-17A to its receptors (IL-17RA, IL-17RC) on target cells of different types. Therefore, we conducted co-staining of CD3+ and IL-17RA, and no overlapping signals were observed on non-lesional, leading edge or lesional biopsies (Fig. 6B). This result indicate that IL-17A molecules, when they are receptor-bound, will most likely not be found on CD3+ T cells, which explains why only a minority of IL-17A staining co-localized with CD3 in Fig. 6A.

## Discussion

Although vitiligo does not have any clear signs of clinical inflammation, it has been established that most cases of non-segmental vitiligo contain a microscopic inflammatory infiltrate [2]. Infiltrating T cells have been found in peri-lesional vitiligo skin, and circulating auto-antibodies and auto-reactive CD8+ cytotoxic T cells that recognize melanocyte antigens were detected in the sera of a high proportion of vitiligo patients [39]. Th1 responses, as characterized by IFN-γ, have been established in vitiligo. T cells expanded from peri-lesional vitiligo skin show a predominately type 1 cytokine profile (i.e. IFN-γ and TNF-α) [12]. The treatment of vitiligo by using IFN-γ inhibitors has also given positive therapeutic responses [14].

A recent advance in our understanding of T cells in autoimmune diseases has been the identification of the Th17 subset. Th17 cells are a distinct lineage of proinflammatory T helper cells that are induced in the presence of IL-6/IL-21, TGF-ß and IL-1ß and expanded under the stimulation of IL-23 [40], [41], [42]. Th17 cells have attracted wide interest as they have been implicated in many inflammatory diseases that were previously only linked to Th1 responses, including rheumatoid arthritis, psoriasis, multiple sclerosis and Crohn's disease [43], [44], [45], [46], [47]. However, not all immune disorders or inflammatory diseases involve an active population of Th17 cells. In the skin, for instance, psoriasis contains a prominent Th17 population [19], [22], but atopic dermatitis has a very minimal Th17 component [48]. In vitiligo, there has been very limited data on whether and how Th17 cells participate in the disease pathogenesis. In a recent study, Basak and co-workers endeavored to compare serum levels of IL-17 between healthy control and vitiligo patient groups. They reported decreased serum TGF-ß levels in vitiligo patients, but a quantitative comparison of serum IL-17 levels between the healthy control and vitiligo patient groups was lacking [20]. In this study, we provided direct tissue evidence for Th17 involvement in vitiligo, manifested by elevated IL-17A mRNA levels and the presence of IL-17A+ T cells in the leading edge of vitiligo biopsies.

Th1 and Th17 cells are often colocalized in autoimmune environments such as psoriasis and multiple sclerosis [22], [49]. It has been proposed that Th1 and Th17 cells collaboratively contribute to human autoimmune diseases. In a psoriasis study, IFN-γ has been shown to be a potent promoter of IL-17+ T cell trafficking, induction and function in humans [50]. In a mouse model, adoptively transferred Th17 polarized cells were able to mediate destruction of advanced B16 melanoma and induce vitiligo, but this therapeutic effect was critically dependent on IFN-γ production, whereas IL-17A and IL-23 depletion had little impact [51]. Our study shows that in leading edge vitiligo biopsies, there is an active Th17 component in addition to a Th1 component. The interplay between Th1 and Th17 populations in vitiligo remains an intriguing avenue for future exploration.

Interleukin 17 can be produced by both CD4+ and CD8+ T cells, as well as natural killer cells and natural killer T cells [43]. In this study, we could not obtain shave biopsies from vitiligo patients to phenotype IL-17A producing T cells by FACS, but we do observe IL17A+ T cells in the dermal area of leading edge vitiligo biopsies, as well as CD8+ cells infiltrating the basal layer of the epidermis. We also see significant colocalization of IL-17A and IL-17 receptor A in leading edge vitiligo skin. It has been reported that vitiligo patients possess high frequencies of circulating CD8+ T lymphocytes specific for Melan-A [52], and melanoma patients who went through Melan-A specific CD8+ T cell infusion immunotherapy demonstrated melanocyte loss in regions of normal skin [10].

IL-1ß is a key cytokine for the development of Th17 cells [37], and it is activated in the inflammasomes formed by NOD-like receptors (e.g. NALP-1, NALP-3) [53]. NALP1 is widely expressed at low levels, but is present at a high level in immune cells, particularly T cells and Langerhans cells [54], which may explain the high IL-1ß levels in leading edge vitiligo biopsies. One of the consequences of cytokine-orchestrated inflammation is apoptosis. In addition to CD8+ cytotoxic T cell-mediated killing, melanocyte loss in the leading edge of vitiligo skin may result in part from increased synthesis and release of IL-1ß, and the accompanying apoptotic microenvironment at the dermal-epidermal junctions.

Th17 cells are antigen restricted, and therefore the development of autoimmune T cells would require antigen presentation by dendritic cells. In theory, both Langerhans cells and dermal dendritic cells could present antigens to T cells. Langerhans cells appeared to be activated in leading edge and depigmented skin based on their high HLA-DR expression and their location in the epidermis could lead to direct contact with melanocyte processes or cellular antigens. Alternatively, inflammatory CD11c+ DCs that invade the epidermis might also capture melanocyte antigens. As already recognized, inflammatory dermal dendritic cells may stimulate Th17 cell proliferation through their production of IL-23 [19]. In vitro data suggest that IL-23 also provides survival signals for already differentiated Th17 cells to strengthen the Th17 phenotype [55]. About half of CD11c+ dermal dendritic cells in the skin are CD1c+, and the number of these increased significantly in leading edge vitiligo biopsies. This is very different from psoriasis where CD1c+ cells are not increased, but inflammatory CD11c+ CD1c− DCs are seen to expand in a dramatic manner. Moreover, it has been discovered that CD11c+ dermal DCs express perforin and granzyme B as well as exhibit cytotoxic activity against tumor cells [56]. It is possible that in addition to presenting melanocyte-specific self-antigens, CD11c+ dermal DCs at the leading edge of vitiligo skin are directly involved in the killing of melanocytes.

In summary, our findings established an activated Th17 axis in vitiligo. The capture and presentation of melanocyte related antigens by different sets of dendritic cells in the skin is an interesting problem that needs to be addressed in future studies. Our observation that Langerhans cells contain activated inflammasomes and that dermal CD11c+ DCs subsets are elevated could indicate involvement of both epidermal and dermal DC populations. This is a particularly interesting problem since Langerhans cells and dermal DCs may have differing abilities to activate Th1, Th2 and Th17 cell populations in humans [57].

## Materials and Methods

### Subjects and skin samples

This study was approved by The Rockefeller University Institutional Review Board. Twenty patients with non-segmental vitiligo were enrolled in the study. Informed written consents were obtained from all patients before their participation. The study was performed with strict adherence to the Declaration of Helsinki Principles. The patients were of 18 years of age or older, and the demographics of these patients are listed in Supplementary Material Table S2. These patients had not undergone any systemic therapies (including methotrexate, etretinate, PUVA or cyclosporine) at least 4 weeks prior to entering the study. In addition, no treatment with topical steroids, calcineurin inhibitors and/or vitamin D analogs were allowed for these patients at least 4 weeks prior to entering the study. Pregnant or lactating women were excluded from this study. For each volunteer, six-millimeter punch biopsies were obtained of lesional (clinically depigmented), leading edge (border between clinically depigmented and pigmented skin) and non-lesional skin (clinically pigmented). Subsequently, samples were frozen in OCT (Sakura, Torrance, CA, U.S.A.) and kept at −80°C for immunohistochemistry.

### Immunohistochemistry

Frozen sections were taken from all of the skin biopsies and were stained with hematoxylin (Fisher Scientific, Pittsburgh, PA, U.S.A.) and eosin (Shandon, Pittsburgh, PA, U.S.A.). For immunohistochemistry, frozen sections were blocked with 10% normal horse serum, and endogenous peroxidases were quenched by incubation with diluted hydrogen peroxide (1∶10 dilution of 3% hydrogen peroxide). Sections were incubated overnight at 4°C with primary mouse monoclonal antibodies. Biotin-labeled horse anti-mouse antibodies were used for secondary binding, and thereafter the signals were amplified with avidin-biotin complex (Vector Laboratories, Burlingame, CA, U.S.A.). Subsequently, the sections were developed using chromogen 3-amino-9-ethylcarbazole (Sigma-Aldrich, St Louis, MO, U.S.A.).

### Immunofluorescence staining

Vitiligo depigmented, pigmented and leading edge skin sections (*n* = 3) were fixed with acetone and blocked in 10% normal goat serum (Vector Laboratories) for 30 minutes. Primary antibody was incubated overnight at 4°C and amplified with the appropriate secondary antibody: goat anti-mouse IgG1 conjugated with Alexa Fluor 568 (Invitrogen/Molecular Probes, Eugene, OR) for 30 mins. For colocalization, sections were then costained overnight with a second antibody: Langerin, HLA-DR, CD3, or IL-17/IL-17RA, and amplified with the appropriate goat-anti mouse IgG2b and IgG2a secondary antibody conjugated with Alexa Flour 488, respectively. Images were acquired using appropriate filters of a Zeiss Axioplan 2 widefield fluorescence microscope fitted with a Plan Neofluar20 x×0.7 numerical aperture lens and a Hamamatsu Orca ER-cooled charge-coupled device camera, controlled by METAVUE software (MDS Analytical Technologies, Downington, PA).

### Antibodies

Table S1 in Supplementary Material lists the sources of antibodies and their concentration for immunohistochemistry and immuofluorescence stainings.

### Reverse transcriptase-polymerase chain reaction (RT-PCR)

Skin samples were frozen in liquid nitrogen and RNA was extracted from homogenized tissue using the RNeasy Mini Kit (Qiagen, Valencia, CA, U.S.A.) and genes were amplified using EZ PCR core reagents, primers and probes (Applied Biosystems, Foster City, CA, U.S.A.). Sequences of primers and probes used in this study were as follows: (IL-17A: Assay ID Hs00174383_m1, IFN-*γ*: Assay ID Hs00989291_m1, IL-1ß: Assay ID: Hs00174097_m1, IL-4 Assay ID: Hs00174122_m1). RT-PCR were performed on Applied Biosystems Prism 7700 Sequence Detector using extracted RNA from vitiligo skin biopsies according to the manufacturer's directions and as previously established [18], [58], [59]. The samples were amplified by using the following thermal cycler conditions: 2 min at 50°C; 30 min at 60°C; 5 min at 95°C; 40 cycles of 15 sec at 95°C followed by 60 sec at 60°C. To make semi-quantitative measurements and comparisons on relative gene expression, each gene expression data were normalized against the house keeping gene hARP (human acidic ribosomal protein), and analyzed with the Applied Biosystems Sequence Detection Systems software version 2.3.

### Cell counts

NIH *Image* program (http://rsb.info.nih.gov/nih-image/; NIH *Image* version 6.1) allowed for manual counting at 20× magnification of total number of stained cells per mm of frozen section.

### Statistical Analysis

Nonparametric Freidman's *t*-tests and two-tail paired t-tests were used to compare cell counts per mm of paired lesional, leading edge and non-lesional skin. Results were interpreted as significant at *P* values less than 0.05, as trends and tending to significance at *P* values less than 0.1. SEM were displayed in all the bar graphs.

## Supporting Information

Figure S1 **CD3 and CD4 double stainings on vitiligo biopsies.** The majority of CD3+ cells are also CD4+ (>60%). In the skin, CD3−/CD4+ population will include dermal dendritic cells; CD3+/CD4− cells are also dectected, indicating a mixed CD4+ and CD8+ T cell infiltrate in vitiligo. (CD3 antibody: BD Biosciences Cat No. 347340; CD4-FITC antibody: BD Biosciences 340133).
(TIF)

Figure S2 **CD3 and CD8 double staining on vitiligo biopsies.** CD3+/CD8+ cells are found in non-lesional, leading edge and lesional vitiligo skin, showing that CD3+ T cells contain a mixture of CD4+ and CD8+ T cells.
(TIF)

Figure S3 **CD83 staining on vitiligo biopsies.** Scattered CD83+ cells with dendritic morphology were found in the upper dermis and epidermis of vitiligo biopsies, including non-lesional, leading edge and depigmented. Some CD83+ cells are located in the epidermis, consistent with LC maturation.
(TIF)

Figure S4 **Positive controls for antibodies used in identifying Langerhans cells and dermal DC subsets.** Before studying Langerhans cells and dermal DCs in vitiligo skin biopsies, all antibodies were tested on psoriasis lesional and non-lesional skin for their reactivity and specificity. Their staining patterns on psoriatic skin were consistent with data published in previous reports from this lab [32], [33].
(TIF)

Figure S5 **NALP-1 and Langerin double staining on vitiligo biopsies.** More NALP-1 positive cells are observed in leading edge vitiligo biopsies. Almost 30% of Langerin+ cell are also NALP-1 positive, whereas in NL, LS or normal skin (data not shown), only 5–10% of Langerin+ cells were also NALP-1 positive.
(TIF)

Figure S6 **IL-17A blocking experiment on psoriatic and normal skin.** (A) IL-17A staining on lesional, nonlesional psoriatic skin and biopsies from normal healthy volunteers (antibody was applied at a dilution of 1∶500). (B) IL-17A antibody was diluted at 1∶500 and incubated at room temperature with recombinant human IL-17A (R&D Systems Cat. No. 317-ILB) for two hours at an Ab to Ag molar ratio of 1∶10. The IL-17A anibody and rhIL-17A mixture was applied to three groups of skin biopsies, and staining was performed in parallel with samples in panel A. After blocking with rhIL-17A, no staining was seen across the three groups of samples except for red precipitates of Ab/Ag complexes. (C) IgG1 isotype control on lesional, non-lesional psoriatic skin and normal skin.
(TIF)

Table S1 **Sources of antibodies and their working conditions.**
(DOCX)

Table S2 **Patient Demographics.**
(DOCX)
