# Supplementary figures and images for: Th17 Cells and Activated Dendritic Cells Are Increased in Vitiligo Lesions
Source: PLoS One. 2011 Apr 25;6(4):e18907. doi: 10.1371/journal.pone.0018907 (PMC3081835; doi:10.1371/journal.pone.0018907)

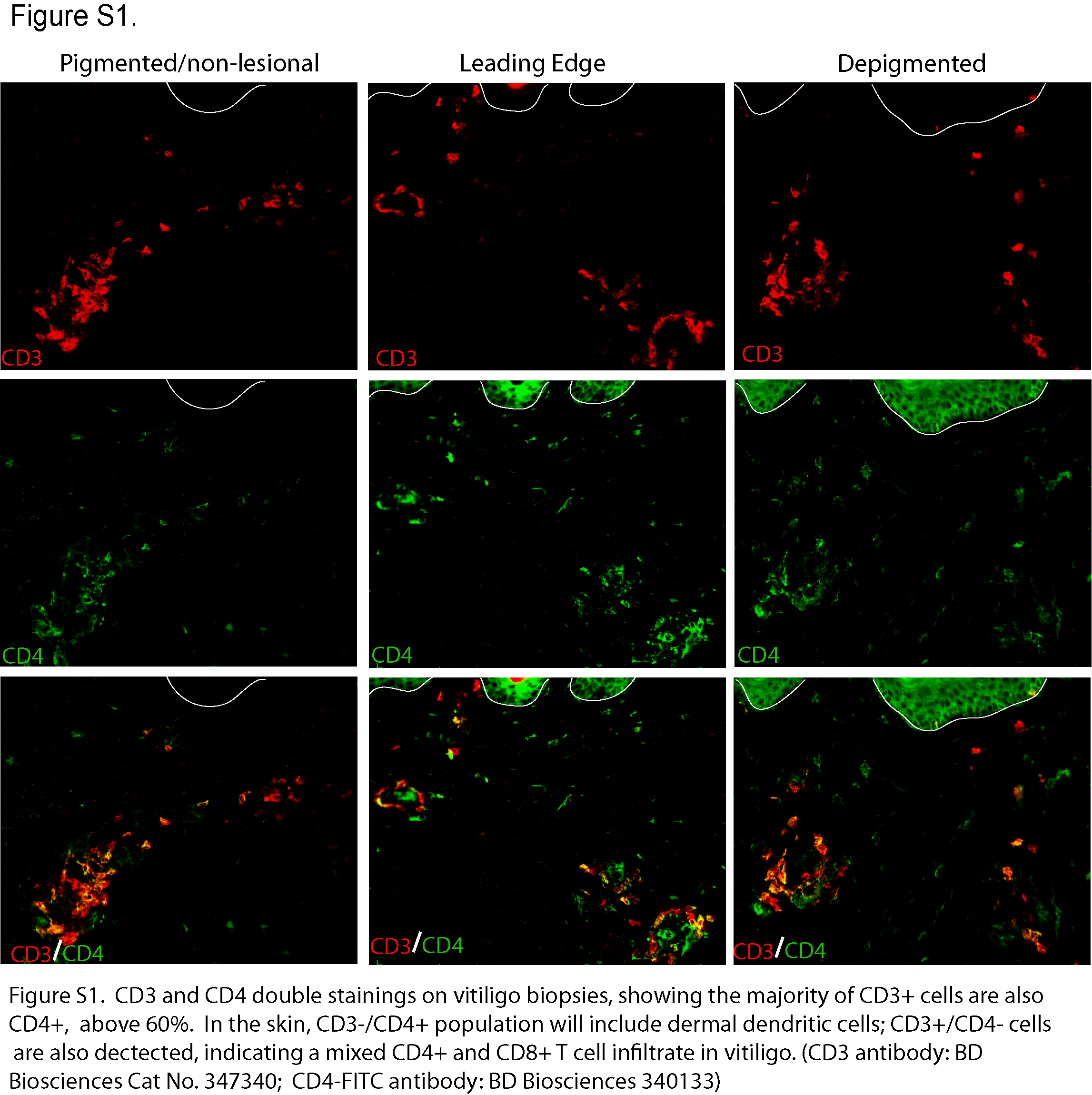

Supplement: Figure S1 — CD3 and CD4 double stainings on vitiligo biopsies. The majority of CD3+ cells are also CD4+ (>60%). In the skin, CD3−/CD4+ population will include dermal dendritic cells; CD3+/CD4− cells are also dectected, indicating a mixed CD4+ and CD8+ T cell infiltrate in vitiligo. (CD3 antibody: BD Biosciences Cat No. 347340; CD4-FITC antibody: BD Biosciences 340133). (TIF) [file pone.0018907.s001.tif]

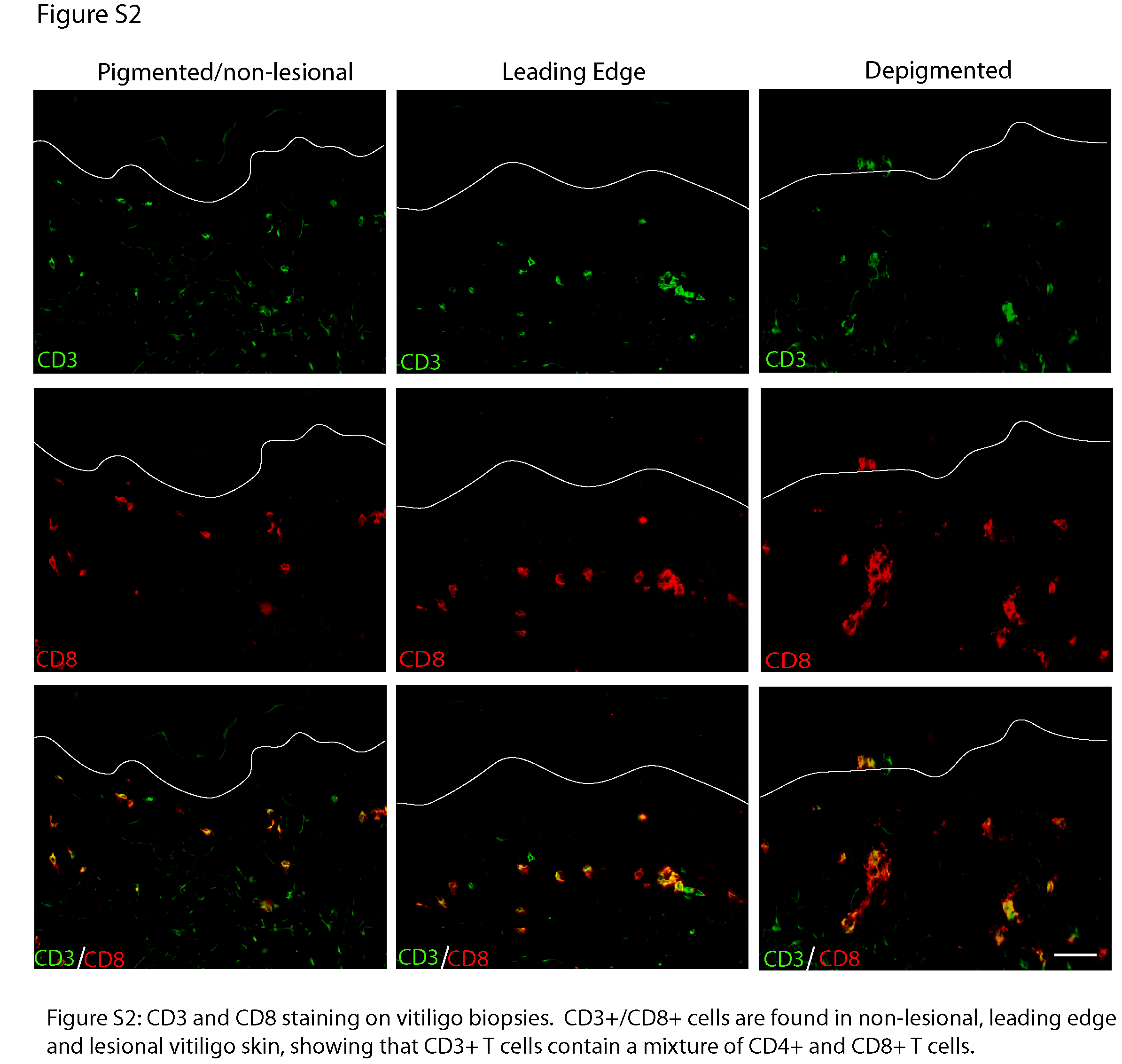

Supplement: Figure S2 — CD3 and CD8 double staining on vitiligo biopsies. CD3+/CD8+ cells are found in non-lesional, leading edge and lesional vitiligo skin, showing that CD3+ T cells contain a mixture of CD4+ and CD8+ T cells. (TIF) [file pone.0018907.s002.tif]

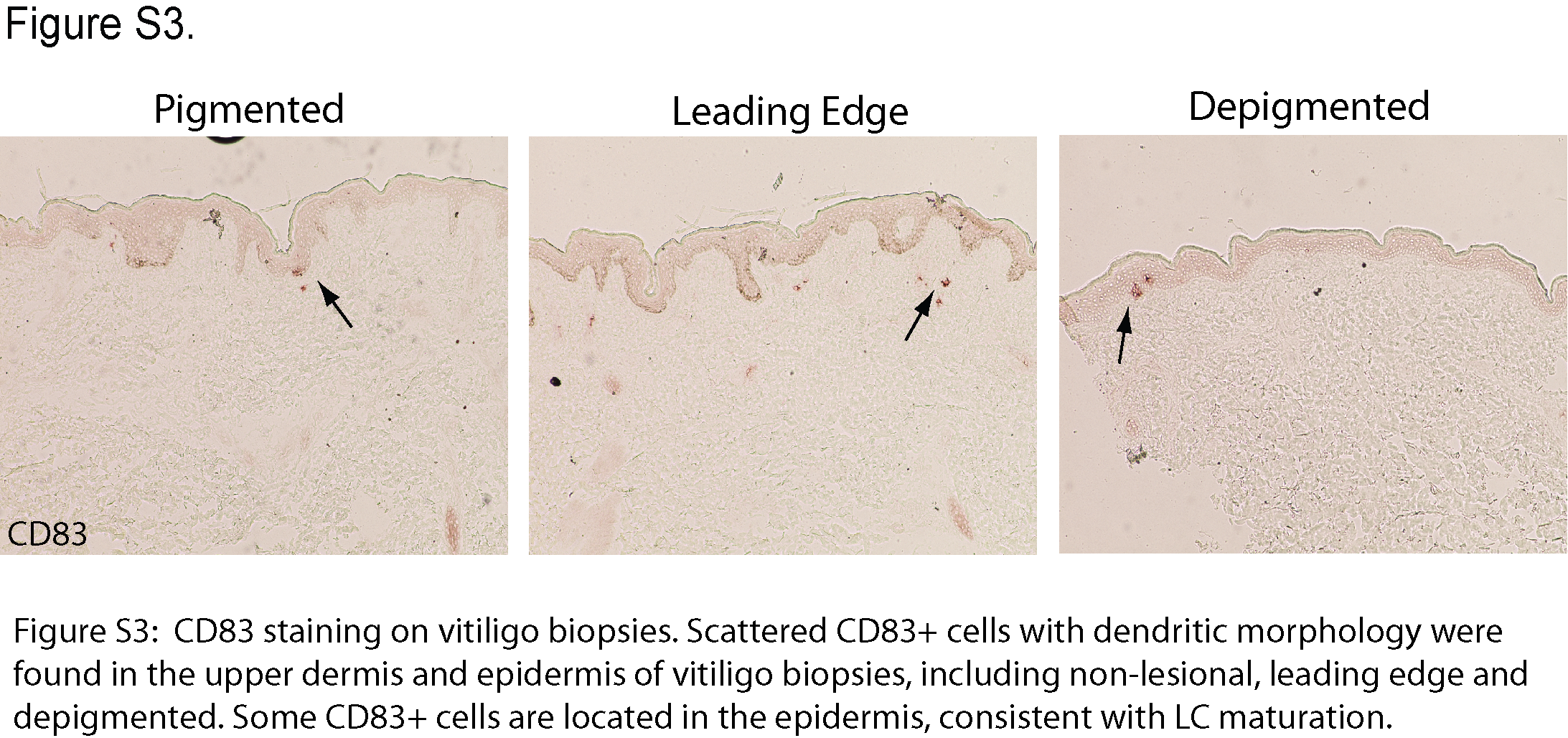

Supplement: Figure S3 — CD83 staining on vitiligo biopsies. Scattered CD83+ cells with dendritic morphology were found in the upper dermis and epidermis of vitiligo biopsies, including non-lesional, leading edge and depigmented. Some CD83+ cells are located in the epidermis, consistent with LC maturation. (TIF) [file pone.0018907.s003.tif]

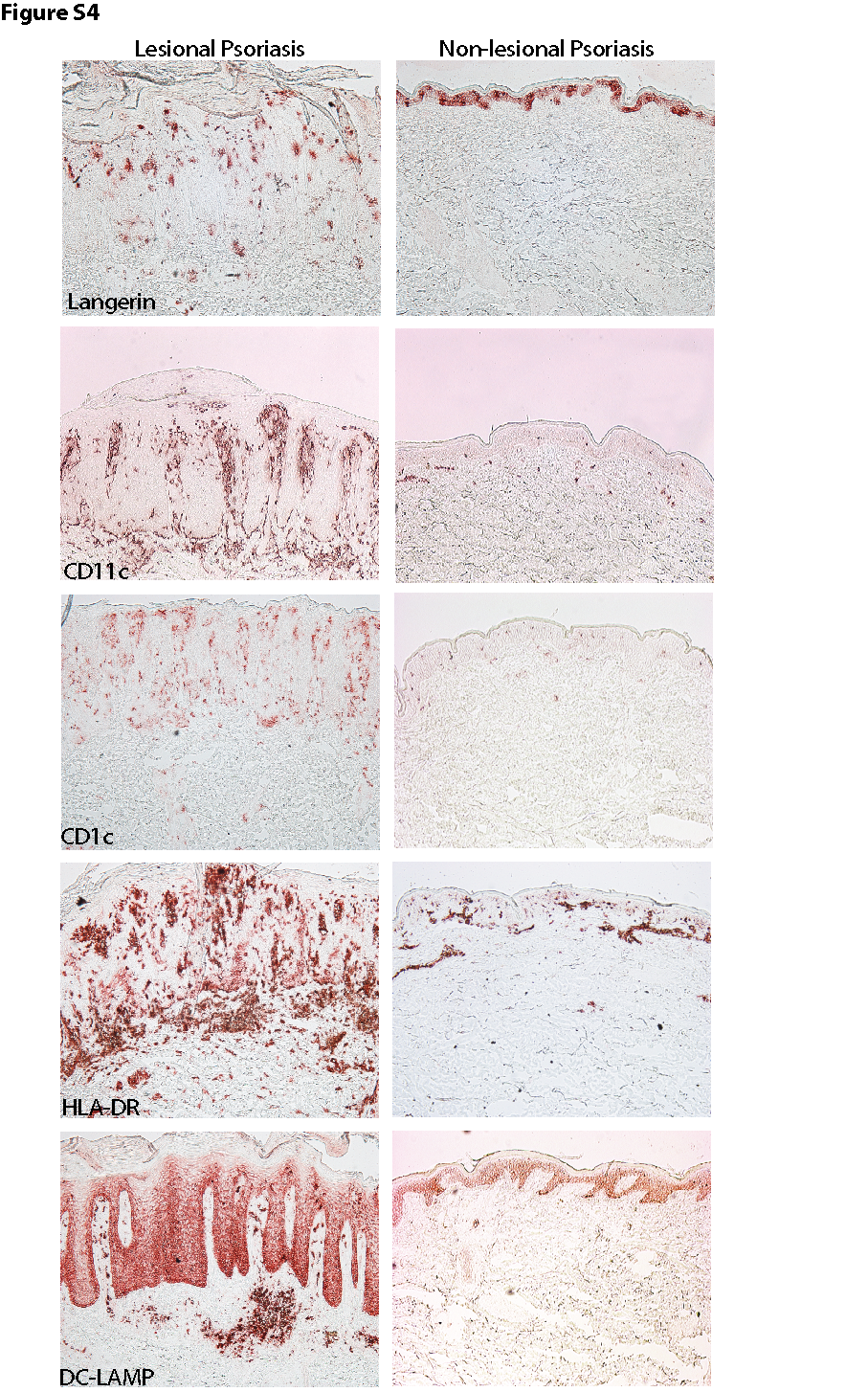

Supplement: Figure S4 — Positive controls for antibodies used in identifying Langerhans cells and dermal DC subsets. Before studying Langerhans cells and dermal DCs in vitiligo skin biopsies, all antibodies were tested on psoriasis lesional and non-lesional skin for their reactivity and specificity. Their staining patterns on psoriatic skin were consistent with data published in previous reports from this lab [32], [33]. (TIF) [file pone.0018907.s004.tif]

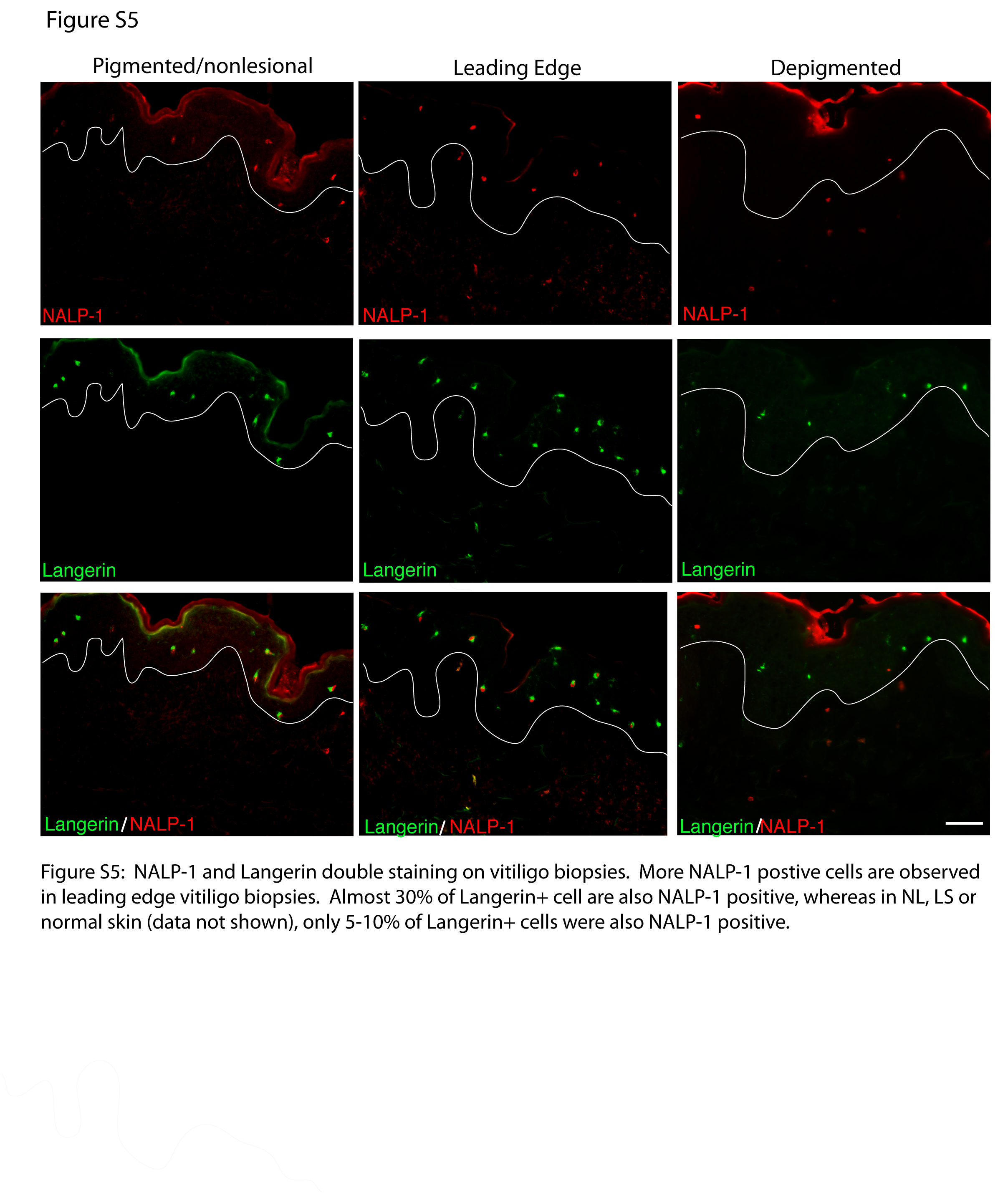

Supplement: Figure S5 — NALP-1 and Langerin double staining on vitiligo biopsies. More NALP-1 positive cells are observed in leading edge vitiligo biopsies. Almost 30% of Langerin+ cell are also NALP-1 positive, whereas in NL, LS or normal skin (data not shown), only 5–10% of Langerin+ cells were also NALP-1 positive. (TIF) [file pone.0018907.s005.tif]

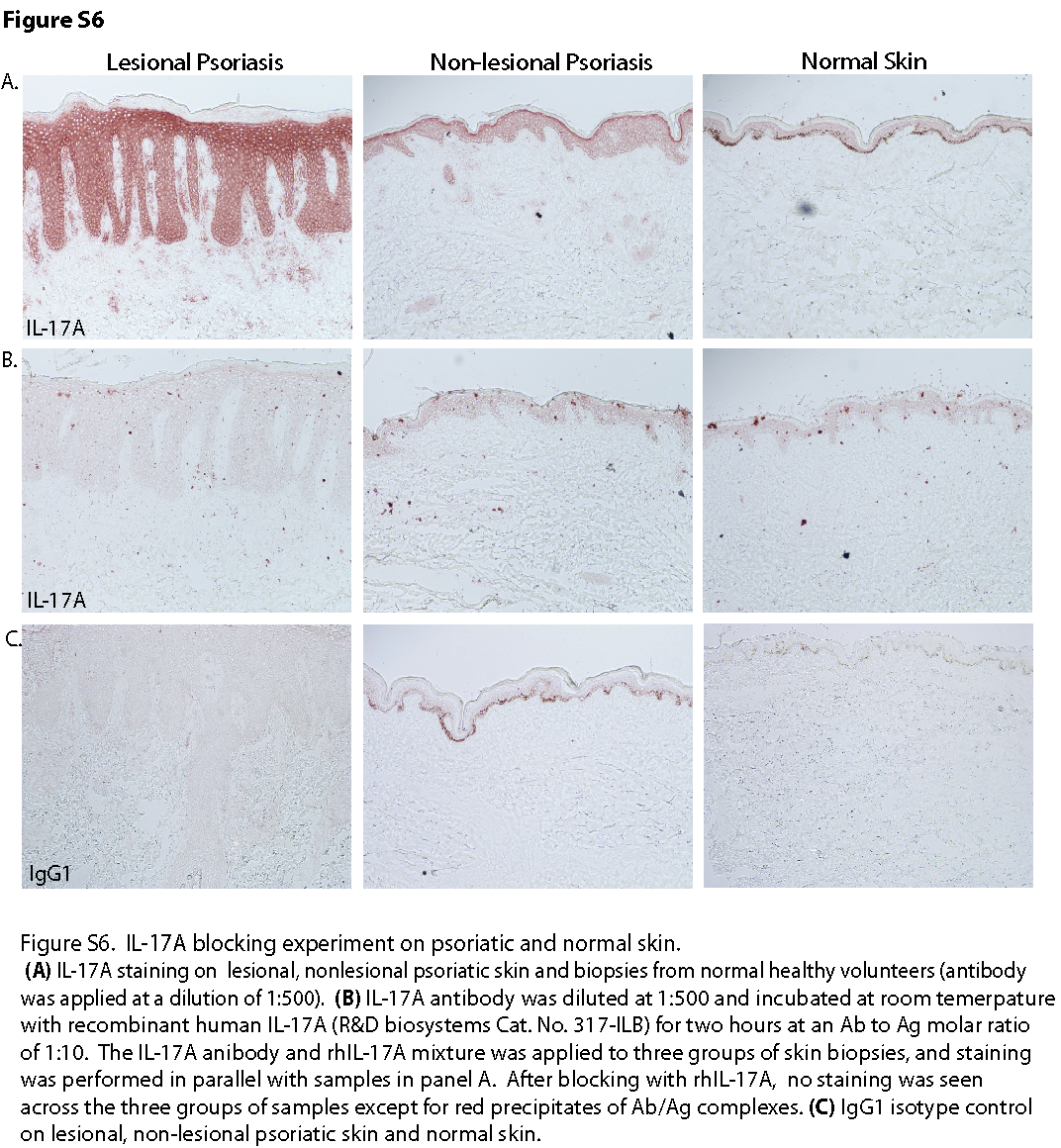

Supplement: Figure S6 — IL-17A blocking experiment on psoriatic and normal skin. (A) IL-17A staining on lesional, nonlesional psoriatic skin and biopsies from normal healthy volunteers (antibody was applied at a dilution of 1∶500). (B) IL-17A antibody was diluted at 1∶500 and incubated at room temperature with recombinant human IL-17A (R&D Systems Cat. No. 317-ILB) for two hours at an Ab to Ag molar ratio of 1∶10. The IL-17A anibody and rhIL-17A mixture was applied to three groups of skin biopsies, and staining was performed in parallel with samples in panel A. After blocking with rhIL-17A, no staining was seen across the three groups of samples except for red precipitates of Ab/Ag complexes. (C) IgG1 isotype control on lesional, non-lesional psoriatic skin and normal skin. (TIF) [file pone.0018907.s006.tif]
