# Supplementary material for: Th17 Cells and Activated Dendritic Cells Are Increased in Vitiligo Lesions
Source: PLoS One. 2011 Apr 25;6(4):e18907. doi: 10.1371/journal.pone.0018907 (PMC3081835; doi:10.1371/journal.pone.0018907)
Supplement: Table S1 — Sources of antibodies and their working conditions. (DOCX) [file pone.0018907.s007.docx]

| **Table S1. Sources of antibodies and their working conditions** | | | | |  |  |
| --- | --- | --- | --- | --- | --- | --- |
| **Antigen** | **Vendor** | **Catalog No.** | **Clone** | **Isotype** | **Dilution** | **Application** |
| Melan-A | Novocastra | NCL-MelanA | A103 | IgG1 | 1:100 | IHC |
| CD3 (FITC) | BD Biosciences | 349201 | SK7 | IgG1 | 1:100 | IF |
| CD3 | BD Biosciences | 555335 | UCHT-1 | IgG1 | 1:100 | IHC |
| CD8 | BD Pharmingen | 555631 | HIT8a | IgG1 | 1:100 | IHC |
| CD1c/BDCA-1 | Miltenyi Biotec | 130-090-695 | AD5-8E7 | IgG2a | 1:100 | IHC |
| CD11c | BD Biosciences | 550375 | B-ly6 | IgG1 | 1:100 | IHC |
| CD207/Langerin | Beckman Coulter | IM3449 | DCGM4 | IgG1 | 1:100 | IHC |
| CD207/Langerin | Abcam | ab49730 | 12D6 | IgG2b | 1:100 | IF |
| DC-LAMP | Immunotech | PN IM3448 | 104.G4 | IgG1 | 1:100 | IHC |
| HLA-DR (FITC) | BD Biosciences | 340857 | L243 | IgG2a | 1:100 | IHC |
| HLA-DR (FITC) | BD Biosciences | 347363 | L243 | IgG2a | 1:100 | IF |
| NALP-1 | Santa Cruz Biotechnology | sc-58550 | Nalpy1-4 | IgG1 | 1:100 | IF |
| IL-17RA | AMGEN | 67071-94 | M202 | IgG2a | 1:100 | IHC/IF |
| IL-17A | R&D Systems | MAB3171 | 41802 | IgG1 | 1:500 | IHC/IF |
