# Supplementary material for: Th17 Cells and Activated Dendritic Cells Are Increased in Vitiligo Lesions
Source: PLoS One. 2011 Apr 25;6(4):e18907. doi: 10.1371/journal.pone.0018907 (PMC3081835; doi:10.1371/journal.pone.0018907)
Supplement: Table S2 — Patient Demographics. (DOCX) [file pone.0018907.s008.docx]

| **Table S2. Patient Demographics** | | | |  |  |
| --- | --- | --- | --- | --- | --- |
| Patient No. | Age | Sex | Ethnicity | Skin areas involved | Years since disease onset |
| 1 | 58 | F | Caucasian | 75% | 30 |
| 2 | 28 | F | Caucasian | 12% | 5 |
| 3 | 55 | F | Caucasian | 60% | N/A |
| 4 | 43 | M | Caucasian | 10% | 3 |
| 5 | 22 | F | Caucasian | 8% | 6 |
| 6 | 36 | M | Asian | 5% | 30 |
| 7 | 50 | F | Caucasian | 20% | 4 |
| 8 | 42 | M | African | 30% | 3.5 |
| 9 | 56 | F | African | 70% | 25 |
| 10 | 37 | F | Hispanic | 30% | 1 |
| 11 | 48 | F | African | 57% | 3 |
| 12 | 44 | F | African | 30% | 4 |
| 13 | 22 | F | Caucasian | 8% | 10 |
| 14 | 32 | F | Asian | 40% | 13 |
| 15 | 35 | M | African | 70% | 10 |
| 16 | 60 | M | Caucasian | 20% | 30 |
| 17 | 26 | F | Caucasian | 25% | 6 |
| 18 | 37 | M | Indian | 70% | 20 |
| 19 | 41 | F | Caucasian | 30% | 30 |
| 20 | 54 | F | Caucasian | 20% | 12 |
